# Semiconducting Polymer Dots for Point-of-Care Biosensing and In Vivo Bioimaging: A Concise Review

**DOI:** 10.3390/bios13010137

**Published:** 2023-01-14

**Authors:** Sile Deng, Lingfeng Li, Jiaxi Zhang, Yongjun Wang, Zhongchao Huang, Haobin Chen

**Affiliations:** Department of Biomedical Engineering, School of Basic Medical Sciences, Central South University, Changsha 410013, China

**Keywords:** polymer dots, biosensors, fluorescent probes, semiconducting polymers, molecular imaging

## Abstract

In recent years, semiconducting polymer dots (Pdots) have attracted much attention due to their excellent photophysical properties and applicability, such as large absorption cross section, high brightness, tunable fluorescence emission, excellent photostability, good biocompatibility, facile modification and regulation. Therefore, Pdots have been widely used in various types of sensing and imaging in biological medicine. More importantly, the recent development of Pdots for point-of-care biosensing and in vivo imaging has emerged as a promising class of optical diagnostic technologies for clinical applications. In this review, we briefly outline strategies for the preparation and modification of Pdots and summarize the recent progress in the development of Pdots-based optical probes for analytical detection and biomedical imaging. Finally, challenges and future developments of Pdots for biomedical applications are given.

## 1. Introduction

Nanomedicine is the study of the application of nanoparticles in the field of biomedicine, and it has made progress in medical diagnosis and treatment, including biosensing, tissue engineering, medical imaging, cell tracking, drug transporting and cancer optical therapy [1,2,3,4]. Generally, biosensors can specifically detect analytes to provide physiological information in a fast and accurate way, and point-of-care testing has become a medical trend, as it greatly facilitates patient self-monitoring of health [5,6,7,8]. Apart from biosensing applications, biological imaging helps to visualize the internal structures or enables functional imaging for disease diagnosis, and multimodal imaging combines several imaging methods to integrate the respective signal containing different aspects of biological information for a more comprehensive diagnosis and accurate treatment [9,10]. With the development of materials and principles, biosensing and bioimaging technologies have received considerable attention due to their advantages of high resolution, real-time and non-invasiveness [11,12,13]. However, since the properties of the materials could exert influence on the sensitivity and accuracy of biosensing and optical applications, traditional small-molecule organic dyes suffer from inherent weakness such as short lifetime, poor photostability and low absorption, which limit further biomedical applications [14,15,16].

Nanomaterials with better properties and performance have been developed and widely used in the biological field [17,18,19]. Nanomaterials used to constitute biosensors have great properties and performance due to their unique nanoscale and easily modifiable characteristics, which benefit the energy transfer. On the other hand, nanomaterials for contrast agents contribute to better penetration depth and conversion efficiency, resulting in higher-quality imaging [20,21,22]. Typical luminescent nanomaterials include quantum dots (Qdots) [23], carbon dots [24], upconversion nanoparticles (UCNPs) [25], aggregation-induced emission (AIE) dots [26] and polymer dots (Pdots) [27]. In particular, Pdots have demonstrated the utilization of optical and biosensing applications in recent years, such as super-resolution imaging [28,29], fluorescence imaging [30,31] and disease-related marker detection [32,33]. Pdots are organic nanoparticles assembled from polymer chains with π-conjugated systems, and the nanoscale size endows Pdots with unique properties, which have attracted extensive attention. According to the definition given by Wu and Chiu [34], the Pdots, specifically considered as a part of semiconducting polymer nanoparticles (SPNs), are nanoparticles consisting of hydrophobic semiconducting polymers with a volume or weight fraction more than 50% and a diameter generally less than 50 nm, sometimes the particles size can be less than 30 nm. Pdots have shown characteristics of large absorption cross section, high brightness, good photostability, low toxicity and various forms of existence and modification, which are the basis of fluorescence probes for complex biological applications, typically for fluorescence-based biosensing and bioimaging [35,36,37].

**Figure 1:**
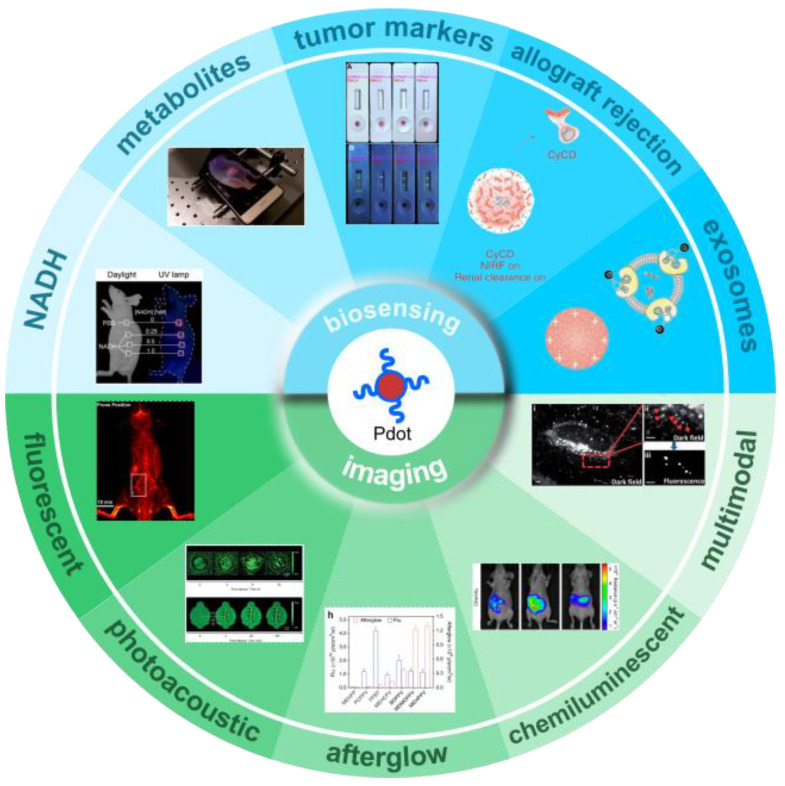
Semiconducting polymer dots for biosensing and imaging.

This review focuses on the fundamental content and recent advances of Pdots in biosensing and bioimaging applications. The preparation and properties of Pdots are briefly introduced. Modification and functionalization are basic and crucial parts of practical applications, which are related to the attachment of functional groups to the surface of nanoparticles. Thus, several surface modification methods are also introduced. Many Pdots have been presented for in vitro biosensing applications and therapy applications, such as ion sensors [38], reactive oxygen species sensors [39], nucleic acid assays [40,41], enzymatic activity assays [42], photodynamic therapy [43], photothermal therapy [44], gene therapy [45] and chemotherapy [46], which are referred to in recent reviews [47,48,49]. Herein, we focus on the latest reported Pdots for point-of-care biosensing and in vivo imaging (Figure 1). Pdots point-of-care biosensors, applied to disease-related-metabolites assays, nicotinamide adenine dinucleotide (NAD) sensing, tumor markers quantification and cancer diagnostics, are detailed to demonstrate their great potential in biosensing and transducing techniques. Then, Pdots used as optical probes in bioimaging, such as fluorescence imaging, photoacoustic imaging (PAI), afterglow imaging, chemiluminescence imaging and multimodal imaging, are highlighted. The properties and biomedical applications of the Pdots summarized in this review are listed in Table 1. In the end, we share the challenges and perspective in this field.

## 2. Semiconducting Polymer Dots

This section may be divided by subheadings. It should provide a concise and precise description of the experimental results, their interpretation, as well as the experimental conclusions that can be drawn.

### 2.1. Methods of Preparation

**Figure 2:**
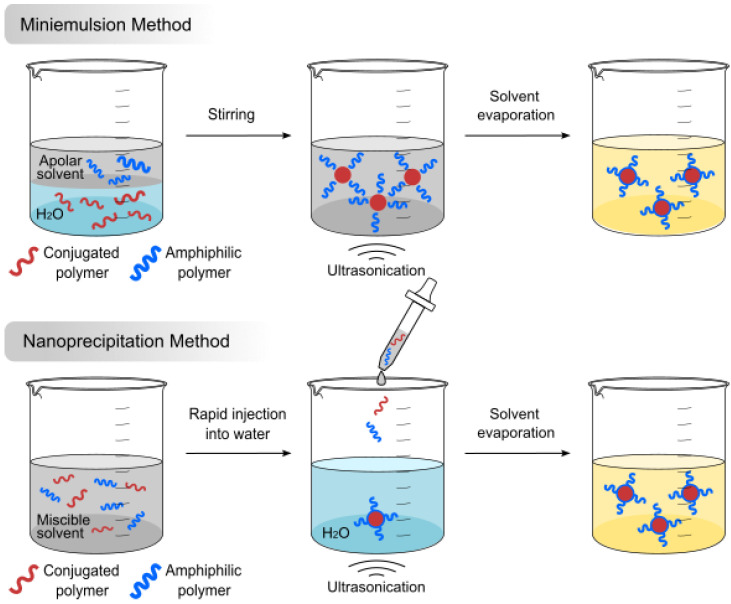
The miniemulsion and nanoprecipitation methods of Pdots.

As different preparation methods generate the Pdots with different sizes and performance, it is critical to choose the corresponding method to obtain suitable size and properties according to various application scenarios. The main preparation methods include the direct polymerization method, miniemulsion method, nanoprecipitation method and self-assembly method. Direct polymerization, referring to the preparation of Pdots from low molecular weight monomers by chemical reactions, offers a wide range of options for size and structure since it also applies to the polymers that are insoluble in organic solvents [68]. Miniemulsion and nanoprecipitation methods dissolve conjugated polymers in organic solvents and interact with water [69,70]. The self-assembly method requires stirring of the solution to mix conjugated polymers and reagents for functionalization. In this part, nanoprecipitation and miniemulsion methods are mainly illustrated (Figure 2).

During the preparation process of the miniemulsion method, the conjugated polymers or monomers to be polymerized are dissolved in a water-immiscible organic solvent [71]. Under vigorous sonication or stirring, it forms microemulsion droplets with aqueous solutions containing surfactants. Finally, stable and uniformly-dispersed Pdots are obtained by removing the organic solvent. In particular, the surfactants are used to avoid aggregation of microemulsion droplets. The concentration of polymers and surfactants in the mixed solution can affect the size of Pdots.

In the nanoprecipitation method, conjugated polymers and amphiphilic polymers are dissolved in a water-miscible organic solvent. Then, the mixed solution is rapidly injected into water under vigorous sonication, and the nanoprecipitation occurs during this process. Pdots with great water dispersibility are obtained by removing the organic solvent. The biggest difference between the miniemulsion and nanoprecipitation methods is the solvent. The nanoprecipitation method uses a water-miscible organic solvent such as tetrahydrofuran (THF), while the miniemulsion method uses a water-immiscible organic solvent such as chloroform. Typically, both methods use surfactants or amphiphilic polymers to increase the yield of nanoparticles. The size of the Pdots depends on the concentration of conjugated polymers in the organic solvents, which ranges from 5 to 50 nm, while the miniemulsion method often gives larger Pdots (larger than 40 nm). In addition, different kinds of amphiphilic polymers can realize different modifications for Pdots in the process of preparation. Liu’s group fabricated uniform Pdots by a microfluidic-assisted nanoprecipitation process with a coaxial microfluidic glass capillary mixer [72]. Wu’s group used the nanoprecipitation method to prepare functional Pdots with carboxyl groups on the surface for further bioconjugation [73]. Further, they combined photo-crosslinking technology to prepare Pdot-based nanocavities, nanoellipsoids, triangular nanorings and nanowires [74,75,76].

### 2.2. Properties and Performance

**Figure 3:**
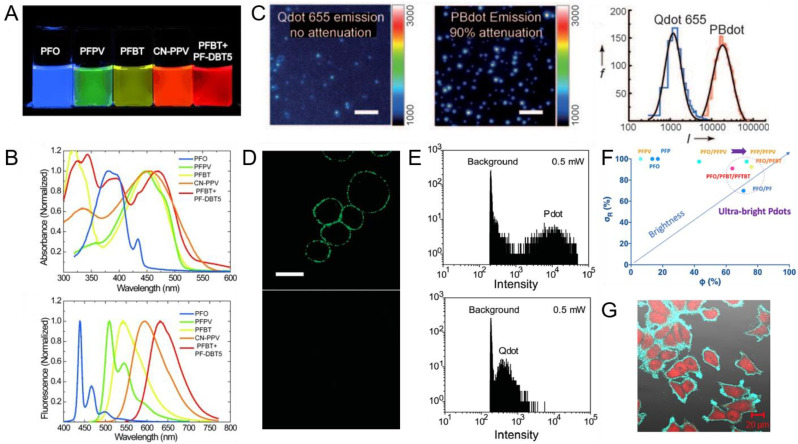
The properties of Pdots. (**A**) A photograph of various Pdots emitted by ultraviolet light. (**B**) Absorption and emission spectra of various Pdots. Reproduced from Ref. [34] with permission. (**C**) Single-particle images and intensity distributions of Qdot 655 and PBdots. Reproduced from Ref. [81] with permission. (**D**) Fluorescence imaging of MCF-7 cells incubated with anti-EpCAM primary antibody and Pdot-lgG conjugates. The bottom panels show the imaging of cells incubated with Pdot-lgG alone. (**E**) Fluorescence intensity distributions for Pdot-streptavidin-labeled MCF-7 cells and Qdot 565-streptavidin-labeled MCF-7 cells. Reproduced from Ref. [82] with permission. (**F**) Ultrabright FRET-based Pdots with simultaneously high absorption cross section and quantum yield. (**G**) Combined fluorescence microscopy images of MCF-7 cells labeled with PEP/PFPV Pdot-streptavidin and biotinylated primary antibody. Reproduced from Ref. [83] with permission.

The critical factors to evaluate the quality of fluorescent probes are absorption cross section, quantum yield, emission rate and photostability. Absorption cross section is used to describe the ability of Pdots to absorb a photon of a particular wavelength and polarization. Studies have shown that the peak absorption cross section of single particles (15 nm in diameter) is about 10–100 times of CdSe Qdots [77]. Moreover, another key property, called quantum yield, is the ratio of the number of photons emitted to the number absorbed; the typical value is below 40% due to aggregation-induced self-quenching [78], and high quantum yield can reach 50–80%. It is generally considered that the brightness of fluorescent molecules depends on the product of absorption cross section and quantum yield. Photostability is assessed by the photobleaching quantum yield calculated from the ratio of photobleaching photons number to the photons absorbed number. Typical photobleaching quantum yield ranges from 10^−4^ to 10^−6^ [79]. Additionally, different kinds of Pdots have been proven to have low toxicity, thus Pdots are widely used in biological applications [80]. Several relevant research results about properties of Pdots are given in Figure 3.

The improvement of properties is a constant proposition in biological applications of Pdots. Recently, Zhang et al. reported fluorescence resonance energy transfer (FRET)-based Pdots with both large absorption cross section and high quantum yield [83]. By choosing acceptors that had a greater spectral overlap with donors or mixing different kinds of Pdots to create cascade FRET Pdots, they obtained ultrabright blue-, green- and red-emitting Pdots that were among the brightest Pdots reported in the visible region. In other examples, Kuo et al. found that the photostability of Pdots can be improved by adding 4-(2-hydroxyethyl)-1-piperazineethanesulfonic acid (HEPES) or 2-(N-morpholino)ethanesulfonic acid (MES) buffer to quench photoinduced radicals, which aided long-term cell tracking in biological imaging [84]. Chang et al. also designed low-toxic cycloplatinated Pdots, used as a photocatalyst to strengthen the photocatalytic efficiency and stability [85].

### 2.3. Surface Modification and Biological Functionalization

#### 2.3.1. Encapsulation Method

Silica encapsulation is widely used for surface modification, as other functional groups could be easily attached to silica, which encapsulates particles in a 2–3 nm shell [86,87]. This method greatly promotes biological functionalization of Pdots. However, the silica shell is possible to hydrolyze in biological environments, and the amino groups used to stabilize silica-encapsulated Pdots (10–20 nm in diameter) also cause nonspecific adhesion between Pdots and the cell surface. Another way for surface modification is to encapsulate Pdots using poly (lactic-co-glycolic acid) (PLGA) [88,89] or phospholipids [90,91], which increases nanoparticle stability and reduces nonspecific adhesion. However, the size of nanoparticles modified by PLGA (230–270 nm) and phospholipids (80–100 nm) is too large to apply at the cellular and subcellular levels. Furthermore, the low concentration of fluorescent polymers eventually limits the brightness of nanoparticles, which causes the failure of the encapsulation method to take full advantages of the Pdots.

#### 2.3.2. Amphiphilic Polymer Coprecipitation Method

Chiu’s group developed some effective functionalization methods [34,82]. They pre-added amphiphilic polymers in organic solvents, and then prepared Pdots by nanoprecipitation. In this process, amphiphilic polymers covered the surface of the hydrophobic nanoparticles, and their hydrophobic ends were randomly bound to hydrophobic Pdots, while the hydrophilic ends were exposed to water. As a result, Pdots with hydrophilic groups were formed to covalently link biomolecules for biological conjugation and functionalization. For example, an amphiphilic polymer, polystyrene-polyethylene glycol-carboxyl (PS-PEG-COOH), was used for surface modification of Pdots [82]. The average diameter of the product was about 15 nm, and more than 80% of the constituents were significantly effective fluorophores. These research results indicated that this strategy can generate efficient nanoparticle probes, since neither the size nor fluorescent properties of Pdots were affected.

**Figure 4:**
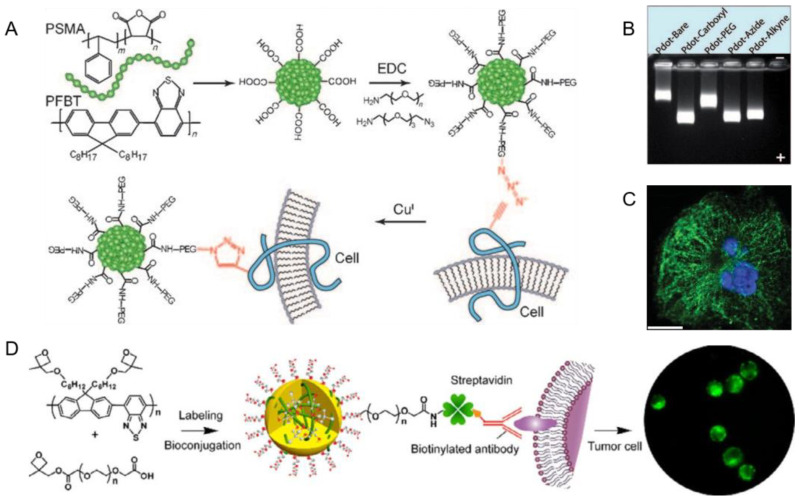
The functionalization methods of Pdots. (**A**) Pdots coprecipitated with amphiphilic polymer PSMA for bioorthogonal labeling via click chemistry. (**B**) Gel electrophoresis of Pdots with various surface functional groups using a 0.7% agarose gel. Reproduced from Ref. [73] with permission. (**C**) Fluorescence microscopy image of microtubules in HeLa cells labeled with cross-linked Pdot-streptavidin. Reproduced from Ref. [93] with permission. (**D**) PEGylated and carboxyl-functionalized Pdots for bioconjugation for specific cellular targeting. Reproduced from Ref. [95] with permission.

Wu et al. used another amphiphilic polymer, poly (styrene-co-maleic anhydride) (PSMA), to realize biological functionalization [73]. The hydrophilic ends were hydrolyzed in an aqueous environment to form Pdots with carboxyl groups, which facilitated further subsequent bioorthogonal labeling by click chemistry (Figure 4A). Dynamic light scattering (DLS) and transmission electron microscopy (TEM) measurements showed the typical image and hydrodynamic diameter (~15 nm) of functionalized Pdots. Among different functional groups on the Pdots surface, the carboxyl-functionalized Pdots had a significant increase in mobility in the gel electrophoresis (Figure 4B).

#### 2.3.3. Direct Functionalization

In the above modification methods, functional molecules are non-covalently linked to Pdots, which is the main reason for functional molecules falling from the surface of Pdots, which ultimately affects the performance of functionalized Pdots. To overcome these drawbacks, Zhang et al. developed an alternative direct functionalization method, in which Pdots covalently link functional groups [92]. They synthesized conjugated polymers with different percentages of carboxyl groups and used them directly to prepare functionalized Pdots to avoid extra surface modification procedures. Moreover, they found that the degree of functionalization influences the stability and performance of Pdots. In addition, the low carboxylic acid group density (2.3%) brings the greatest properties, including fluorescence brightness, colloidal stability, non-specific absorption and compact internal structure. Yu et al. reported a cross-linking strategy to form functionalized Pdots with enhanced labeling efficiency and sensitivity for cellular assays (Figure 4C) [93]. In addition, Zhang and Chen et al. developed facile strategies with an optical stimulus to covalently link polyethylene glycol and/or carboxyl functional groups to the Pdots (Figure 4D) [94,95], and demonstrated effective bioconjugation of these functionalized photocross-linkable Pdots for specific cellular labeling.

## 3. Application of Pdots Biosensors in Point-of-Care Diagnostics

Point-of-care diagnostics are analytical assays outside the laboratory in order to ensure the convenience of fast testing for target analytes in patients with the same accuracy as laboratory tests. Recently, Pdots-based biosensors have been used for point-of-care diagnostics due to their superior photophysical properties and efficient energy transfer or electron transfer.

FRET has facilitated tremendous advances in biosensing for point-of-care applications. FRET is a phenomenon in which energy is non-radiatively transferred from a donor fluorophore to an acceptor fluorophore, where the fluorescence of the acceptor is emitted while the fluorescence of the donor is quenched. Nanoscale Pdots enable efficient energy transfer, as the efficiency of FRET strongly depends on the distance between donor and acceptor [96]. Additionally, FRET in Pdots biosensors could enhance the brightness of Pdots and obtain high absorption cross section and great photostability [97]. Specific dyes are added into Pdots to realize corresponding FRET-based biosensing [98], which could be applied to detect various metabolites and physiological information [95], including reactive oxygen species [99,100,101], pH [102], temperature [103,104] and metal ions [105,106]. In this review, Pdots-based biosensors for biomolecule detection are mainly discussed.

### 3.1. Nicotinamide Adenine Dinucleotide (Oxidized Form: NAD^+^; Reduced Form: NADH)

NADH plays an extremely important role in redox reactions as a coenzyme in enzyme-catalyzed reactions associated with physiological processes [107]. The variation of NAD^+^ and NADH concentration is one of the manifestations of diseases such as cancer, epilepsy and Parkinson’s disease [108,109,110]. Biosensors, realizing the convenient and accurate measurement of NAD^+^ and NADH concentrations in point-of-care, can promote research advances in the diagnosis of related diseases.

**Figure 5:**
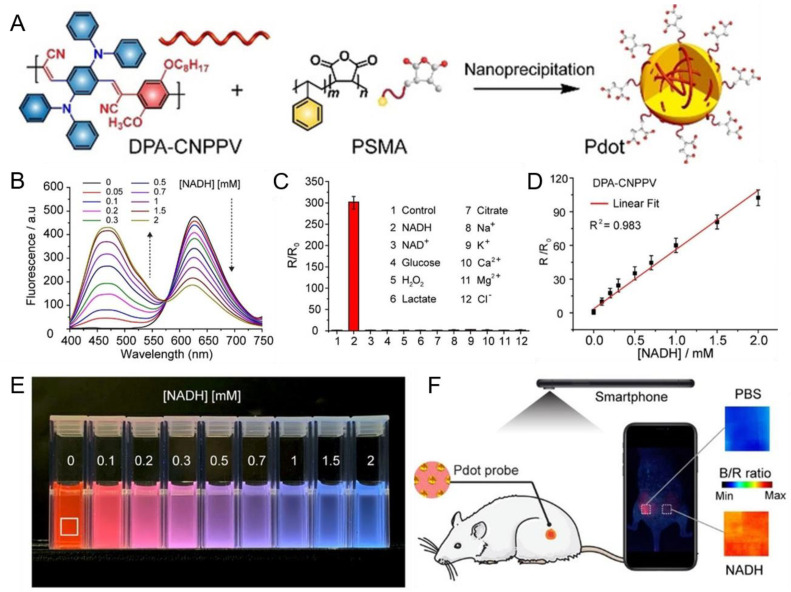
DPA-CNPPV Pdots for reversible ratiometric NADH sensing. (**A**) The preparation of DPA-CNPPV Pdots by nanoprecipitation. (**B**) Emission spectra of DPA-CNPPV Pdots with 0–2 mM NADH. (**C**) Selectivity of DPA-CNPPV to NADH versus 10 other analytes. (**D**) Calibration curve of DPA-CNPPV Pdots in the range of 0–2 mM NADH. R = I_458 nm_/I_627 nm_; R_0_ = R in the absence of NADH. (**E**) DPA-CNPPV Pdots at 0–2 mM NADH with excitation at 365 nm. (**F**) Ratiometric imaging taken by smartphone for NADH in vivo measurement. DPA-CNPPV were injected into two locations of mice with or without NADH (0.1 μmol). Heatmap images of blue/red-channel intensities ratios are shown on the right, with a high ratio (red) indicating high concentration of NADH. Reproduced from Ref. [50] with permission.

Chen et al. developed a series of Pdots for ratiometric NADH sensing [50], including PFO, PDHF, PFBT, PFBTTBT, PFTBT and DPA-CNPPV Pdots (Figure 5A), which were prepared by the nanoprecipitation method with PSMA. For DPA-CNPPV, the emission maximum was exhibited at 627 nm with excitation at 385 nm. In the presence of NADH, electron transfer from excited DPA-CNPPV to NADH caused fluorescence quenching of DPA-CNPPV Pdots, which was manifested as a decrease in emission intensity at 627 nm. Since NADH in solution had a blue emission (peak at 458 nm), while DPA-CNPPV hardly absorbed in the blue region, there was almost no energy transfer from NADH to DPA-CNPPV. In short, with the increasing concentration of NADH, the emission intensity decreased at 627 nm and increased at 485 nm in the physiological range (0–2 mM, Figure 5B). For other Pdots, for example, the absorption of PFBT in the blue region caused less color contrast, which was not favorable for ratiometric sensing. On the other hand, the NADH-sensitive DPA-CNPPV Pdots had shown great performance in terms of photostability, response time, selectivity (Figure 5C) and reversibility.

A DPA-CNPPV Pdots probe used for NADH measurement in vivo showed the potential for point-of-care testing. The emission proportion of the DPA-CNPPV Pdots probe changed with the increasing NADH concentration, and the true-color photographs were taken by smartphone for further analysis. The blue/red ratio, with a linear response to NADH concentration, was considered as a key parameter for NADH sensing, which was calculated by dividing the true-color photographs into blue and red channels (Figure 5D,E). Applying this protocol to mice (Figure 5F) enabled ratiometric NADH sensing.

### 3.2. Disease-Related Metabolites

Metabolites assays play a key role in early disease diagnosis and management for the reason that its blood levels are closely related to the disease or injuries; for example, phenylalanine in phenylketonuria, glucose in diabetes, tyrosinase in tyrosinemia, glutamate during ischemic strokes, galactose in galactosemia and leucine in maple syrup urine disease. However, it cannot reliably detect most metabolites, hindering disease diagnosis and management [111]. To address this issue, many researchers have developed new methods for stoichiometric-based metabolite detection using Pdots biosensors.

#### 3.2.1. Glucose

According to the International Diabetes Federation, an estimated 8.8% of adults aged 20–79 had diabetes in 2015, and the proportion is expected to rise to 10.4% by 2040 [112]. Diabetes is a metabolic disease with increased concentration of blood glucose. Long-term high blood glucose can cause great damage to the heart, kidneys and nervous system of diabetic patients. Blood glucose management is a vital part of diabetes treatment, including instant sampling detection and dynamic real-time monitoring.

**Figure 6:**
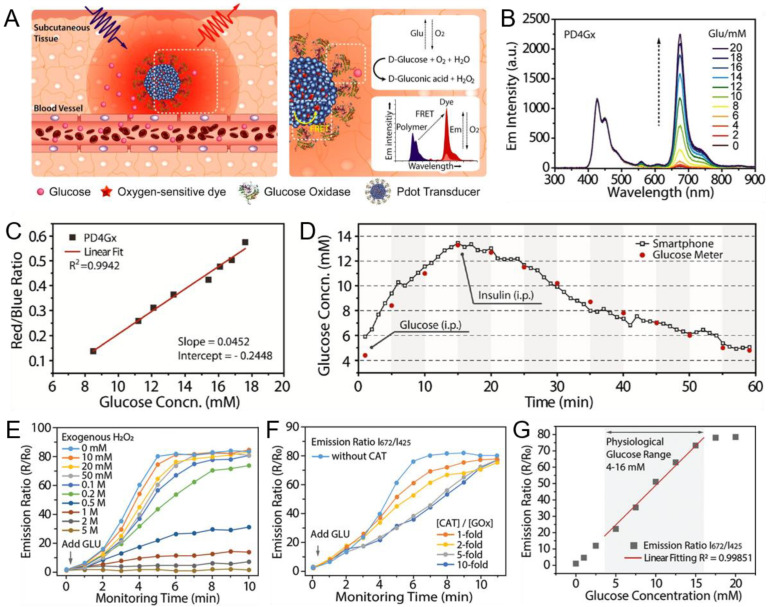
Pdots transducer used for wireless glucose monitoring. (**A**) The design and detection principle of PD4Gx. (**B**) Emission spectra of PD4Gx in a series of PBS solutions with different glucose concentrations. (**C**) Calibration curve by comparing red/blue ratio with standard glucose values. (**D**) Real-time detection for glucose concentration by PD4Gx transducer (black) and commercial glucose meter (red). Reproduced from Ref. [51] with permission. (**E**) Response curves of the Pdot–GOx transducer with different concentrations of exogenous hydrogen peroxide. (**F**) Response curves of the Pdot–GOx/CAT transducer with different CAT/GOx ratios. (**G**) Liner relationship between glucose concentration and emission ratio of Pdot-GOx/CAT with 5-fold CAT/GOx ratio. Reproduced from Ref. [114] with permission.

Recently, functionalized Pdots covalently linked to glucose oxidase (GOx) have been used as biosensors for real-time monitoring due to their excellent properties and outstanding detection results [113]. In the presence of analytes, internal oxygen is depleted to translate the oxygen concentration into a fluorescent (or phosphorescent) signal. Based on this principle, Wu’s group developed an ultrasensitive Pdots transducer that enables wireless glucose monitoring [51]. The transducer was mainly composed of phosphorescent dyes, GOx and Pdots functionalized by PSMA (Figure 6A). PDHF was selected as the light-harvesting host. In addition, the oxygen-sensitive Pd(II) meso-tetra (pentafluorophenyl) porphine (PdTFPP, D4) was doped in PDHF Pdots. The glucose biosensor (PD4Gx) was formed by binding GOx to carboxyl groups on the surface of PDHF. FRET between PDHF and D4 caused changes in emission light in the presence of glucose, since GOx-catalyzed oxidation of glucose causes changes in oxygen levels. Upon ultraviolet (UV) excitation, the blue fluorescence from PDHF Pdots was quenched, and the glucose biosensors showed red phosphorescence from D4. The emission spectrum of solutions with different glucose concentrations are shown in Figure 6B. With increasing glucose concentration, the ratio of blue and red light changed, causing the sensors to emit different colors. During the experiment, they implanted PD4Gx sensors subcutaneously in mice, took photographs of the light-emitting part with a smartphone and found that the luminescent images were obviously altered after glucose infusion. Glucose concentrations were acquired by comparing the calibration curve (Figure 6C) with the emission ratio obtained from true-color image processing. Data from the continuous monitoring of the P4DGx glucose sensors were in good agreement with the discrete measurements from commercial blood glucometers (Figure 6D), indicating that this ultrabright Pdots transducer enabled dynamic real-time wireless monitoring of blood glucose in living mice.

However, there are some drawbacks that can be improved in the above research. Hydrogen peroxide was produced during the process of glucose oxidation, resulting in photobleaching that degraded glucose sensor performance (Figure 6E), reducing enzymatic activity and generating cytotoxicity. Thus, Sun et al. developed their research approach to construct Pdots-based glucose sensors with an enzymatic cascade system (Pdots-GOx/CAT) by adding catalase to the above sensors to rapidly decompose hydrogen peroxide and improve the photostability and biocompatibility of glucose sensors [114]. Figure 6F,G shows the performance of this Pdots-GOx/CAT glucose transducer, which indicates an excellent long-term sensing ability for monitoring glucose. However, the reaction of peroxide decomposition, physical activity and pathological factors changed oxygen levels in tissues, thereby affecting the accuracy of glucose concentration. Therefore, Sun et al. also proposed an assumption to prepare a second oxygen-sensitive Pdots sensor not coupled with GOx, for the purpose of measuring the altered oxygen levels outside the glucose biosensor and further correcting the changes to improve the accuracy of the results [115]. The feasibility of this method had been demonstrated by numerical simulations and in vivo experiments. Other problems arising from implantable Pdots sensors were the aggregation and migration of nanoparticles in subcutaneous tissue, which was possibly caused by the direct implantation of free Pdots, and affected the detection of luminous intensity. Liu et al. proposed an injectable hydrogel implant to disperse Pdots evenly in it. The hydrogel remained at the implant site for one month without migration. The findings suggest that this method can be used for long-term glucose monitoring [116].

#### 3.2.2. Phenylalanine

Phenylketonuria (PKU) is a metabolic genetic disorder that causes a defect in phenylalanine hydroxylase, preventing the conversion of phenylalanine to tyrosine and increasing blood phenylalanine levels [117]. This mechanism typically results in neurological damage in infants and children with PKU. PKU screening for newborns and management of blood phenylalanine levels in PKU patients have received much attention.

Chen et al. designed a metabolite biosensor consisting of NADH-sensitive Pdots and phenylalanine dehydrogenase (PheDH) [118]. On the basis of this biosensor, a paper-based point-of-care assay for blood phenylalanine levels was developed for PKU screening. Phenylalanine underwent oxidation catalyzed by PheDH to form phenylpyruvate and NADH. In the presence of NADH, red fluorescence was quenched and blue fluorescence was emitted. The emission intensities ratio at 458 nm and 627 nm showed an 18.9-fold change in concentration, from 0 to 2400 μM. In the process of the assay, plasma samples were added into test paper with lyophilized buffer containing modified Pdots, NAD^+^ and PheDH. The blood phenylalanine concentrations were calculated from the emission intensity ratio of the blue and red channel. The performance of Pdots biosensors applied to a healthy person (60 μM) and a classic PKU patient (1200 μM) showed a significant difference in the ratio of blue and red channel intensity. Moreover, the error caused by endogenous NADH was corrected by analyzing samples both in the presence and absence of PheDH. The difference in concentrations obtained in the absence and presence of PheDH, respectively, was considered to be the exact blood phenylalanine concentration. The measurement result of plasma samples with phenylalanine were obtained by using a digital camera. Such a system can be promoted to the concentration measurement of any other metabolite oxidized by NAD^+^ or reduced by NADH.

### 3.3. Tumor Markers

**Figure 7:**
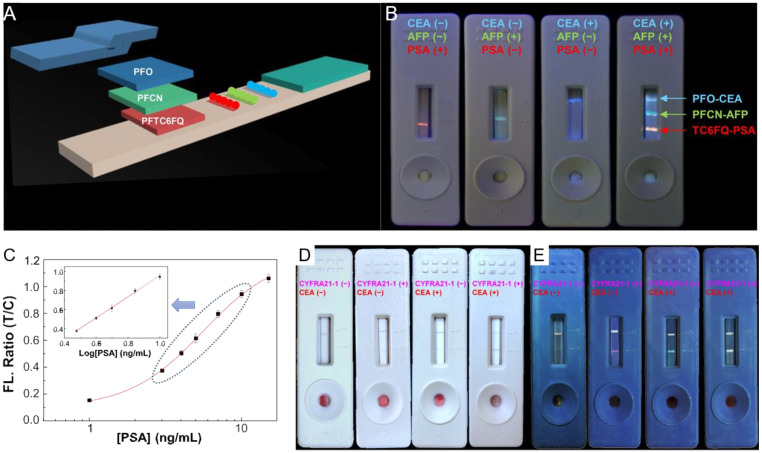
Multiplexed ICTS based on Pdots for detection of CEA/AFP/PSA. (**A**) The structure and principle of multiplexed ICTS for detection of multiplexed tumor markers. (**B**) Photographs of detection results with multiplexed tumor markers concentration (0/0/5, 0/5/0, 5/0/0, 5/5/5 ng/mL). Reproduced from Ref. [52] with permission. (**C**) The calibration curve of the Au650@Pdot immunoassay platform with 3–10 ng/mL PSA. Reproduced from Ref. [119] with permission. (**D,E**) Photographs of Au@Pdot-based test strips with samples containing different concentrations of CYFRA21-1 and CEA obtained under ambient light (**D**) and UV light (**E**). Reproduced from Ref. [120] with permission.

Immunochromatographic test strips (ICTS) have become an important tool for point-of-care testing (POCT) of tumor markers. Especially, ICTS based on multicolor Pdots with excellent fluorescent properties can be used for multiplex detection of target analytes. Chan’s group developed a series of Pdots-based ICTS for the detection of multiple tumor markers [52,119,120]. For the simultaneous detection of prostate-specific antigen (PSA), α-fetoprotein (AFP) and carcinoembryonic antigen (CEA) in a single test strip, Fang et al. developed ICTS based on PF-TC6FQ/PFCN/PFO Pdots, which emit red, green and blue fluorescence, respectively [52]. The test strip consists of an absorbent pad, conjugate pad, sample pad and nitrocellulose membrane. Pdot–antibody conjugates, prepared by conjugation of Pdots functionalized with carboxyl groups and PSA/AFP/CEA antibody, respectively, were added into the conjugate pad of the test strip (Figure 7A). The control lines and test lines in the nitrocellulose membranes were modified with a caption antibody and bare lgG, respectively. The absorbent pad, conjugate pad and sample pad were then sequentially adhered to the nitrocellulose membrane to assemble the test strips. Samples dropped on the sample pad would move due to the capillary force. Regardless of the presence of target tumor markers, Pdot–antibody conjugates connected with the bare lgG in the control line to emit fluorescence to indicate the validity of test strip. In addition, in the presence of target tumor markers, Pdot–antibody conjugates were specifically captured by the corresponding caption antibody in the test line and also emitted the specific fluorescence, which gave the positive results. In the absence of target analytes, no emission can be observed in the test line, which gives the negative results. The multiplexed detection of PSA/AFP/CEA was obtained by direct observation with the naked eye under ultraviolet light, as shown in Figure 7B.

Quantitative detection should be calculated according to the fluorescence intensity ratio of the test line to the control line (T/C). With the increasing of tumor markers concentration, the fluorescence brightness of the test line enhanced, while that of the control line decreased. The reason for this phenomenon was that the number of Pdot–antibody conjugates was certain in a test strip; the more Pdot–antibody conjugates were bound to the test line in the presence of tumor markers, the less were bond to the control line, resulting in the comparison of fluorescence intensity of these two lines. According to the study results, the fluorescence ratio of T/C was linearly related to the log [PSA/AFP/CEA] concentration in the range of 3–15 ng/mL. The limit of detection was two orders of magnitude lower than that of conventional detection methods, which indicated that Pdot-based ICTS was beneficial for early diagnosis, rapid screening or regular monitoring of cancer.

Other types of test strips have been developed to improve the detection performance and expand the range of applications. You et al. used Pdot–Au hybrid nanocomposites, formed from Pdots and Au nanorods, with dual colorimetric and fluorometric readout abilities, for rapid screening (colorimetry) and accurate detection (fluorometry) of PSA [119]. The calibration curve of quantitative performance for PSA of this Au650@Pdot immunoassay platform is given in Figure 7C. A drop of whole blood could realize the detection of PSA, since the plasma was captured by the sample pad, whereas conventional testing requires pretreatment of whole blood. Yang et al. reported another dual-modal Au@Pdot-based immunoassay for the detection of CEA and cytokeratin 19 fragments (CYFRA21-1) in the blood of non-small-cell lung cancer patients [120]. The test line was simultaneously modified by the two corresponding caption antibodies, causing four different types of luminous modals with two luminous lines, representing four detection results (Figure 7D,E). Similarly, this Au@Pdot-based ICTS had good linearity in the quantitative analysis for CYFRA21-1 and CEA detection.

### 3.4. Cancer and Allograft Rejection

Polymer nanoparticles can be used for detecting biomarkers related to cancer and allograft rejection in early diagnosis. An advanced method for in vivo imaging and therapy utilizes the interconversion between nanoparticles and small molecules, since it has lots of advantages such as deeper penetration and broader biodistribution due to the small size of molecules, longer retention at the disease site of nanoparticles formed by the biomarker-activated conversion, fast clearance and specific sensitivity [121,122,123]. Pu’s group recently reported the activatable polyfluorophore nanosensors (APNs) with biomarker-activated renal clearance and fluorescence response for bioimaging and urinalysis, which consisted of protease-reactive peptide brushes, cascaded self-immolative linker and caged fluorophore units [53]. Cathepsin B and granzyme B, corresponding with tumor status and lymphocyte activation in allograft rejection, were chosen to be the biomarkers of APNs. In the intrinsic state, APNs were non-fluorescent and accumulated at the disease site. Then, in the activated state, which was caused by the presence of disease-related biomarkers, the protease released the renal-clearable fluorophore fragments. These fragments were further cleared though the kidneys for fluorescent urine analysis. The high renal clearance efficacy and specificity testing of biomarkers made APNs-based urinalysis superior to other detection methods.

### 3.5. Exosomes

Exosomes, extracellular vesicles containing protein, DNA and RNA from the cells that secrete them, take part in cell communication and are involved in pathogenesis of several diseases, including cancer, neurodegeneration and infections [124]. Therefore, they have recently been used in new assays for disease-related biomarker identification and therapeutic response monitoring. Development of biosensors for exosome detection is significant for early cancer diagnosis. Lyu et al. reported the first near-infrared (NIR) afterglow nanosensor for multiplex differentiation of cancer-related exosomes, which consisted of a complex (ASPNC) formed by an NIR semiconducting polyelectrolyte with a quencher-tagged aptamer [54]. Poly(phenylenevinylene)-based (PPV-based) Pdots were used as the backbone for afterglow luminescence, while an NIR ^1^O_2_ photosensitizer, tetra-phenylporphyrin (TPP), was added into the PPV backbone for red-shift emission and afterglow signal amplification. The side chains on PPV, cationic quaternary ammonium groups, enabled the formation of ASNPC with the black hole quencher 2 (BHQ-2)-tagged aptamer. The fluorescence and afterglow signals of ASPNC were both quenched due to the electron transfer between PPV and BHQ-2. However, in the presence of exosomes, the specific binding between exosomes and the designed aptamer hampers the electron transfer and activates the fluorescence and afterglow signals. In their studies, a comparison of the limit of detection (LOD) and of afterglow signal with fluorescence signal indicated that afterglow detection enabled the minimization of background interference and achieved an LOD two orders of magnitude (~93-fold) lower than fluorescence detection. By orthogonally assaying of five kinds of exosomes at the expression levels of four biomarkers, they showed the recognition ability of this afterglow nanosensor and suggested that the different BHQ-2 targeted aptamers mediated the specific binding of ASPNC for potential orthogonal analysis of multiplex exosomes.

Additionally, Jiang et al. developed a method using exosomes labeled with Pdots for superresolution mapping with location error less than 5 nm and excellent optical adjustable duty cycles [125]. In their studies, the switch-on frequency of Pdots were tuned to obtain the structure of a large number of exosomes within a few minutes. A combination of two Pdots and one fluorophore that conjugated antibodies against three different tetraspanins on a seminal exosome were used for multicolor superresolution mapping to simultaneously achieve high throughput and high imaging quality of three tetraspanins. This method can also be applied to understand the structure of other similar biological vesicles, which promotes the application of vesicles in disease diagnosis.

## 4. Application of Pdots Optical Probes in Bioimaging

Optical imaging plays a critical role in disease diagnosis, which includes fluorescence imaging, PAI, afterglow imaging, chemiluminescent imaging, bioluminescence imaging, multiphoton imaging and harmonic imaging. Traditional materials for bioimaging have limitations such as low brightness, photobleaching and toxicity, while Pdots are considered as potential bioimaging materials due to their high brightness and good biocompatibility [126,127,128].

### 4.1. Pdots for Fluorescence Imaging

Fluorescence imaging enables tumor imaging, cell labeling and targeting, vascular structure imaging, etc. This imaging technology requires no ionizing radiation and enables real-time imaging with targetability and high spatial resolution to provide the exact location and silhouette of the targeted object. In particular, NIR fluorescence imaging allows in vivo fluorescence imaging with deeper penetration in biological tissue and less background fluorescence than visible-light fluorescence imaging. An ideal NIR fluorescent agent applied for targeting optical probes should work in the NIR window (NIR-I at 700–900 nm and NIR-II at 1000–1700 nm) and have great photophysical properties, such as brightness, quantum yield and photostability and sufficient strokes shift [125,126]. Indocyanine green (ICG) and methylene blue (MB) are commonly used as NIR fluorescent agents. However, ICG and MB lack sufficient targetability against tumors and the ability of specific conjugation, with low quantum yield and poor photostability [129,130,131,132]. Thus, NIR fluorescent Pdots have developed as fluorescence probes in fluorescence imaging due to their good targetability and low background fluorescence and have been applied in lymph node localization [57], tumor imaging [58] and cancer cell tracking and imaging [133].

NIR fluorescent Pdots can be used for cell labeling to track cell migration non-invasively. Xiong et al. prepared NIR fluorescent Pdots for long-term tumor cell tracking in vivo [55]. They doped the NIR dye, silicon 2,3-naphthalocyaninebis(trihexylsilyloxide) (NIR775), into poly [2-methoxy-5-(2-ethylhexyloxy)-1,4-phenylenevinylene] (MEH-PPV) to prepare the fluorescent nanoprobes. FRET between MEH-PPV as a donor and NIR775 as an acceptor achieved NIR emission, with the absorption peak at 504 nm dominated by MEH-PPV and the emission peak at 776 nm dominated by NIR775. Additionally, the optimal weight ratio of NIR775 to MEH-PPV (0.012:1) decreased the self-quenching and provided the highest efficiency. In their study, 20 μg of NIR Pdots were used to treat 2 × 10^5^ HeLa cells, and then cells were injected into nude mice. The fluorescence of NIR Pdots remained at 75% after 7 days and 28% after 23 days. According to their research results, NIR Pdots proved to have proper strokes shift for reducing errors caused by background fluorescence, weak cytotoxicity evaluated by CCK-8 assays, long-term labeling ability and photostability, which indicated the potential of NIR Pdots-based nanoprobes in the field of in vivo tumor imaging. Furthermore, Feng et al. developed an ultrasmall Pdots with excellent specificity and fast clearance for targeted tumor cell imaging, suppressing nonspecific cell uptake that limited the targetability and sensitivity of nanoprobes [134].

**Figure 8:**
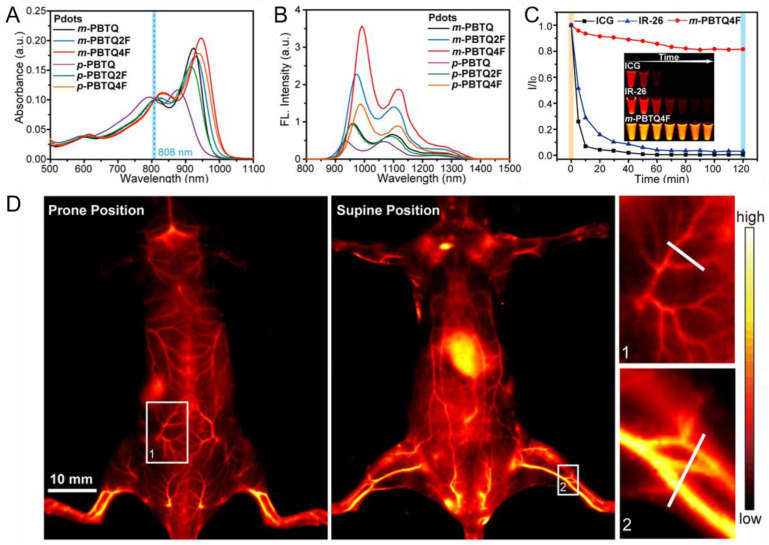
The performance of m-PBTQ4F Pdots for vasculature imaging. (**A**) Absorption spectra of the various Pdots. (**B**) Emission spectra of the various Pdots. (**C**) The photostability under excitation at 808 nm of ICG, IR-26 and m-PBTQ4F in corresponding solvent. (**D**) The NIR-II fluorescent images of C57BL/6 mice injected with 100 mL of m-PBTQ4F into the tail vein in prone and supine positions. Reproduced from Ref. [56] with permission.

Based on the application of NIR fluorescent Pdots for labeling and tracking of targeted cells, in vivo tumor imaging technology has expanded and developed new methods by several improved strategies. Liu et al. developed a fluorination method of Pdots for high brightness in the NIR-II window, which promoted the application of NIR-II probes in brain tumor imaging [56]. They fluoridated PBTQ (m-PBTQ4F) to improve its photophysical properties by using semiconducting polymer synthesized with benzodithiophene (BDT) as a donor and triazole [4,5-g]-quinoxaline (TQ) as an acceptor and changing the quantity and location of the fluorine substituent on the TQ acceptor. The m-PBTQ4F showed excellent fluorescence intensity and photostability compared to ICG and IR26 NIR fluorophores (Figure 8A–C) due to the nanoscale fluorous effect. To assess the quality of m-PBTQ4F used in in vivo fluorescence imaging, they used m-PBTQ4F Pdots for tail-vein injection to show the whole-body vasculature structure of C57BL/6 mice in both prone and supine positions (Figure 8D). In addition, in the prone position, blood vessels in the back were observed clearly, which indicated that m-PBTQ4F Pdots could successfully display in vivo vasculature for tumor fluorescence imaging, since microvascular proliferation and pleomorphic vessels are one of the typical characterizations of malignant brain tumors [135]. The blood vessels were evenly distributed in the normal brain, whereas they were unevenly and chaotically distributed in brain tumors. Therefore, the brain tumors were revealed by vascular structure images obtained from NIR-II fluorescent imaging using m-PBTQ4F Pdots.

### 4.2. Pdots for Photoacoustic Imaging

PAI is a non-invasive biomedical imaging technique that involves the energy conversion from biological tissue. The photoacoustic (PA) contrast agents in the biological tissue absorb energy when they are irradiated by pulsed laser, and then generate ultrasound signals due to transient thermoelastic expansion, which is also referred to PA signals. PA images with high selectivity and penetration depth can be reconstructed by detecting the PA signals containing information about light absorption characteristics. The intensity of PA signal depends on the internal competition of the PA contrast agents between fluorescence emission and non-radioactive heat dissipation. Pdots have been used as PA contrast agents for PAI since Pdots have high photothermal conversion efficiencies and PA intensity [136,137,138,139,140].

#### 4.2.1. Amplification of PA Signal from Pdots

To reduce the toxicity, many design strategies have been developed to amplify the PA signal, which aids to decrease the dosage of PA contrast agents formed by Pdots [141,142]. Adapting the structure of Pdots is one of the design strategies to amplify the PA signal. Guo et al. designed a series of Pdots by using different electron acceptors and planar electron donors, which demonstrated the high photothermal conversion efficiencies and signal-to-background ratio (SBR) of 47 in PAI for tumors in vivo at a depth of 3.2 mm [143]. Dong et al. also prepared PTIGSVS nanoparticles with a high photothermal conversion efficiency of 74% that proved to be a superior PA contrast agent. Another way to amplify the PA signal is to enhance the non-radioactive heat dissipation by strengthening the fluorescence quenching [144]. Lyu et al. developed an intraparticle molecular orbital engineering approach to induce electron transfer with light irradiation, causing enhanced heat production, which increased the PA signal intensity of Pdots by 2.6-fold and maximum photothermal temperature by 1.3-fold [145]. For some Pdots with original faint fluorescence, accelerating the heat dissipation can also enhance the PA signal intensity. Zhen et al. reported that Pdots with a silica layer on the surface simulatively improved the fluorescence and PA brightness due to the higher interfacial thermal conductance between the silica layer and water [146]. Duan et al. also developed CP-IO nanocomposites in which the additional heat production and faster heat dissipation caused by IO nanoparticles resulted in amplification of the PA signal. The above research progress indicates that Pdots for PA contrast agents have potential development space and application prospects for PAI [147].

#### 4.2.2. Pdots for NIR-II PAI

NIR-II PAI usually has higher SBR and penetration depth compared to NIR-I imaging due to reduced light attenuation and lower absorption by biological tissues in the NIR-II window. Recently, Numerous research studies have demonstrated that the Pdots-based NIR-II PA contrast agents have increased SBR and penetration depth, as well as a higher maximum permissible exposure and image resolution [148,149,150,151]. In addition, metabolizable ability related to biotoxicity is another key property of NIR-II PA contrast agents for in vivo PAI, which is an issue to be researched.

**Figure 9:**
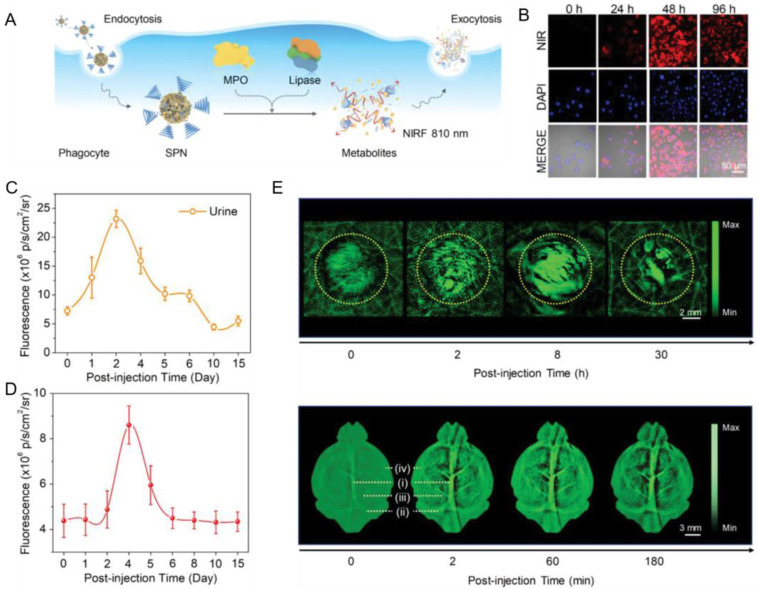
Metabolizable Pdots NIR-II contrast agents for in vivo PAI. (**A**) The degradation and metabolism process of Pdots in phagocytes. (**B**) Confocal images of cells incubated by SPN-PT at different time points. (**C**) Variation of fluorescence intensity with time in the urine of living mice after vein injection of SPN-PT. (**D**) Variation of fluorescence intensity with time in the liver region. (**E**) PA images of a superficial tumor and brain vasculature of injected mice at special time points under irradiation at 1064 nm. Reproduced from Ref. [59] with permission.

Jiang et al. designed a group of metabolizable NIR-II Pdots contrast agents that were easily degraded by relevant enzymes in phagocytes to ultrasmall metabolites, which were cleared out by hepatobiliary and renal excretions after PAI [59]. Three different Pdots (SPN-OT, SPN-PT and SPN-DT) were obtained from three corresponding semiconducting polymers encapsulated into poly(ethyleneglycol)-methyl ether-block-poly(lactide-co-glycolide) (PLGA–PEG) by nanoprecipitation. The obtained Pdots had peak absorption at about 1079 nm and an average hydrodynamic diameter of about 30 nm. The PA spectrum of obtained Pdots ranged from 680 nm to 1064 nm, while the PA amplitude of blood proved to significantly decrease at 1064 nm, indicating these Pdots had increased SBR in PAI due to the background noise from blood, which decreased at 1064 nm in the NIR-II window. SPN-PT was detected for the metabolism and clearance processes as a representative Pdot. Myeloperoxidase (MPO) in phagocytes efficiently degraded semiconducting particles, and lipase in phagocytes catalyzed the hydrolysis of ester linkages of PLGA-PEG. After combined treatment of MPO and lipase, the hydrodynamic diameter of SPN-PT reduced from 30 nm to about 1 nm. The degradation product was fluorescent and then expelled from phagocytes, as shown in Figure 9A. The NIR-II fluorescence signal of degraded products of SPN-PT in the cytoplasm of cells was detected using a confocal laser scanning microscopy (Figure 9B), and the fluorescence was obviously observed after 48 h of incubation, which indicated the degradation in phagocytes was efficient and successful. They then delivered SPN-PT into living mice to study the clearance pathways inside the organism. The fluorescence signal of blood reached a maximum on the first day, then, that of urine dominated by renal clearance also reached a maximum on the second day, as shown in Figure 9C. Subsequently, the signal from the liver reached the top on the fourth day (Figure 9D), and, finally, feces on the fifth day. Such a sequence demonstrated that SPN-PT was successfully degraded in living mice and cleared out though the renal pathway in the beginning and hepatobiliary pathway later. To investigate the NIR-II PA ability of SPN-PT, they used a superficial tumor modal and a deep transcranial brain vasculature modal via vein injection of SPN-PT. The PA amplitude of tumor region and brain vasculature both increased (Figure 9E), making it easier to observe the structure of superficial tumor and brain blood vessels. All the results indicated that these Pdots were efficient NIR-II contrast agents for PAI in vivo and had great biosafety ensured by renal and hepatobiliary clearance.

Men et al. also reported metabolizable highly absorbing NIR-II Pdots for PAI-guided photothermal therapy (PTT), with ultrasmall size at 4 nm, good biocompatibility and photostability, high photothermal conversion efficiency and tumor ablation capability [60]. These metabolizable and ultrasmall Pdots were prepared by a conjugated polymer, Poly([2,5-bis(2-decyltetradecyl)-2,5-dihydropyrrolo [3,4-c]pyrrole-1,4-dione-3,6-dithienyl]-co-[6-(2-ethylhexyl)-[1,2,5]thiadiazolo [3,4-f]benzotriazole-4,8-diyl]) (DPP-BTzTD), and PSMA though the nanoprecipitation method. The particle size revealed by DLS of the obtained DPP-BTzTD S-Pdots was 4 nm, and L-Pdots were 25 nm. The NIR spectra of obtained S-Pdots and L-Pdots showed high absorption in the NIR-II region.

DPP-BTzTD S-Pdots exhibited good photothermal properties. The temperature variation of S-Pdots at different power densities integrated the laser-power-dependency of the photothermal effect. In addition, the rapid temperature increases of S-Pdots at different concentrations also showed the concentration dependency of the photothermal heating effect. The photothermal stability of S-Pdots was assessed by five repeated laser on/off cycles with NIR-II laser irradiation, while the highest temperature was detected within the same range. The photothermal conversion efficiency of S-Pdots was calculated to be 53.1%. Furthermore, the PAI capability of S-Pdots was demonstrated by in vitro phantom tests. The PA signal intensities of S-Pdots were detected at different concentrations from 50 to 200 μg mL^−1^ to show the linear relationship between PA signal intensity and concentration of S-Pdots. The in vivo PAI performance of S-Pdots was inspected by monitoring the PA intensities at the tumor site at designed time points of a 4T1 tumor-bearing nude mice modal. The PA intensity peaked 2 h post injection and stabilized at 24 h post injection, which also revealed the increased metabolizable ability of the S-Pdots. DPP-BTzTD S-Pdots showed the potential utility for NIR-II contract agents with ultrasmall particle size, high photothermal conversion efficiency, rapid excretion and strong PA signal.

### 4.3. Pdots for Afterglow Imaging

In addition to the imaging methods mentioned above, other luminescence imaging including afterglow imaging [152], chemiluminescent imaging [153] and bioluminescence imaging [154] provides different imaging characteristics and advantages. Such methods realize imaging decrease background interference caused by irradiation light in fluorescence imaging, causing higher SBR of images. Pu and co-workers have developed a series of polymer nanoparticles for afterglow imaging. Afterglow materials emit light long after ceasing to provide excitation light. Thus, the fluorescence from afterglow materials and biological tissues can be separated to obtain better images. Miao et al. presented Pdots with a diameter less than 40 nm, emitting luminescence at 780 nm with a half-life of about 6 min. Such afterglow Pdots were demonstrated to be more than 100-fold brighter than inorganic afterglow materials in afterglow intensity, and 127-fold higher than NIR fluorescence imaging in SBR of living mice tumor imaging [61]. Xie et al. reported self-assemble poly(p-phenylenevinylene) derivatives for metastatic tumor afterglow imaging in living mice, which detected the xenograft tumors with volumes of 1 mm^3^ and tiny peritoneal metastatic tumors [62]. These afterglow nanoparticles also showed the potential utility for oxygen partial pressure imaging due to the oxygen-sensitive afterglow property.

### 4.4. Pdots for Chemiluminescent Imaging

Electrochemiluminescent and chemiluminescent Pdots for imaging generate light without light irradiation, which significantly reduces the background interference and photodamage [155]. Zhen et al. developed Pdots doped with naphthalocyanine dye for detection of hydrogen peroxide with intraparticle chemiluminescence resonance energy transfer, realizing the ultrasensitive imaging for hydrogen peroxide in several mouse modals [63]. Moreover, Cui et al. firstly reported Pdots activated by superoxide anion to generate the chemiluminescent signal for in vivo imaging in cancer immunotherapy [64]. Pdots accumulated into tumors and produced the chemiluminescent signal corresponding with the concentration of superoxide anion.

### 4.5. Pdots for Multimodal Imaging

**Figure 10:**
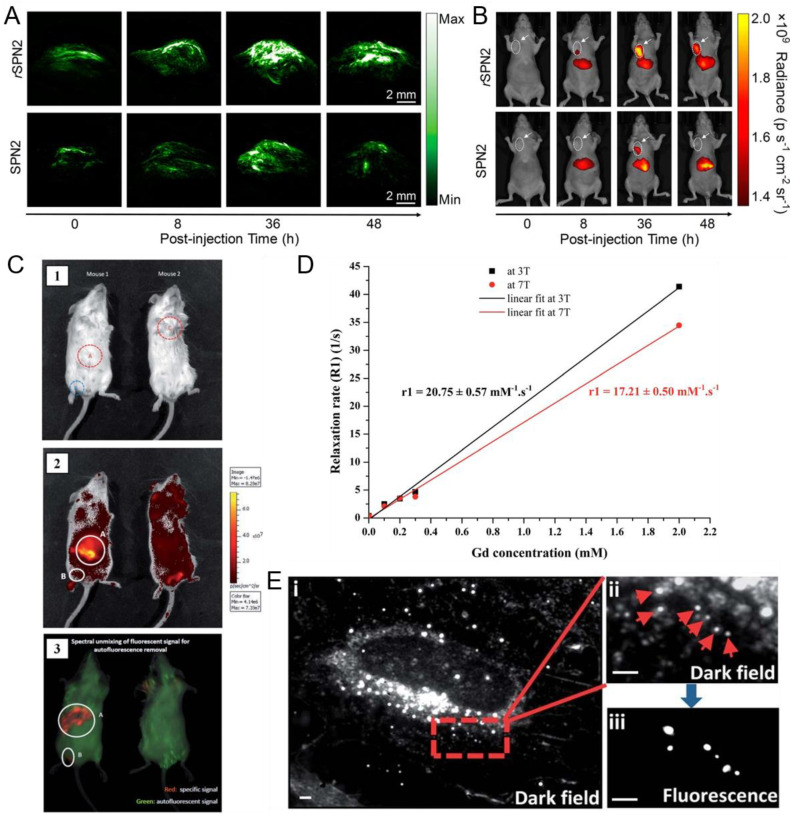
Pdots for multimodal imaging. (**A**) PA images and fluorescence images (**B**) of tumor in living mice injected with rSPNs though the tail vein. Reproduced from Ref. [65] with permission. (**C**) The photograph (1), IVIS image (2) and IVIS processed image (3) of living mice after injection of Gd-SPNs. (**D**) The linear correlation between relaxation rate values (R1) and gadolinium concentration of Gd-SPNs. Reproduced from Ref. [66] with permission. (**E**) Dark field and fluorescence images of Au-NP-Pdots inside mammalian cells. Reproduced from Ref. [67] with permission.

Pdots with dual imaging capabilities have been developed since the multimodal imaging attracts much interest in biological imaging. In relevant studies, the designed Pdots served as fluorescent probes and gave other forms of imaging signals, such as signals in PAI, magnetic resonance imaging (MRI), computed tomography (CT) imaging and singlephoton emission computed tomography (SPECT) imaging to offer the location or physiological information of the detection area for better diagnosis and treatment effects [156,157,158,159,160]. Lyu et al. reported reaction-based Pdots (rSPNs) with a sulfenic acid reactive group (1,3-cyclohexanedione) on the surface [65]. Figure 10A,B shows the PA maximum intensity projection images and fluorescence images of tumors in living mice injected with rSPNs via the tail vein. rSPNs with intense NIR absorption and fluorescence made it possible to achieve fluorescence imaging and real-time PAI for protein sulfenic acids in tumors.

Multimodal Pdots for fluorescence and MRI have also been developed. Hashim et al. reported bifunctional Gd-Pdots prepared by semiconducting polymers, amphiphilic phospholipids and gadolinium-containing lipids, and measured their fluorescence quantum yield, extinction coefficient and MRI T_1_-weighted relaxation times in water [66]. Figure 10C shows the photograph, IVIS image and IVIS processed image of living mice injected with Gd-Pdots, the red fluorescence from Gd-Pdots and green fluorescence from mice were distinguished from each other. The linear correlation between relaxation rate values (R_1_) and gadolinium concentration of Gd-Pdots at 3T and 7T was calculated and plotted in Figure 10D. The fluorescence and magnetic resonance properties of Pdots indicated their potential utility for dual modal fluorescence imaging and MRI.

Additionally, Sun et al. reported small Pdot nanocomposites containing gold nanoparticles to demonstrate the practicality of Pdot-Au nanoparticles for dual-modal imaging involving fluorescence from Pdots and scattering from Au [67]. They used Au passivated by hydrophobic molecules and semiconducting polymers in tetrahydrofuran to prepare Au-NP-Pdots by nanoprecipitation. Au-NP-Pdots inside mammalian cells were imaged by a fluorescence microscope equipped with dark field optics (Figure 10E). Au nanoparticles were useful in long-term tracking and imaging in dark field mode since there was no photobleach. However, the nanoscale cellular organelles strongly scattering light made it difficult to distinguish from Au nanoparticles. In the presence of Pdots, the bright spots in fluorescence mode had corresponding bright spots in dark field mode so that the Au-NP-Pdots that generated both the fluorescence and scattering signals were differentiated from other cellular features.

## 5. Conclusions and Outlooks

Pdots have become an important material for point-of-care testing and in vivo imaging, which greatly assist the diagnosis and treatment of diseases. In this review, several preparation methods are described to demonstrate ways to obtain Pdots with different ranges of particle diameter. In addition, Pdots have excellent photophysical properties and performance, including large absorption cross section, high quantum yield, good photostability and low toxicity. Moreover, recent studies have reported different approaches to further improve these properties. In addition to the size and performance, Pdots can be adapted to special application aims and environments though several modification and functionalization methods. All these methods mentioned in this review indicate that the size, properties and function of Pdots can be tuned for practical applications, suggesting greater utility compared to the traditional materials. For biosensing application, Pdots biosensors based on FRET or electron transfer have been used for the detection of glucose, phenylalanine, NAD and tumor markers, all of which have shown good assay results with convenient, accurate and rapid characteristics. For in vivo bioimaging applications, Pdots can be used as optical probes for fluorescence imaging or as contrast agents for NIR-II PAI, which not only have deeper penetration depth, higher brightness, better resolution and higher contrast efficiency, but also exhibit rapid metabolic capacity relevant for biosafety. Such a large range of applications and outstanding results have proven the significance of Pdots for point-of-care diagnostics and in vivo bioimaging.

Considering the highly homogeneous product landscape, Pdots-based diagnostic techniques have great potential in the sensing and imaging markets. However, Pdots for disease diagnosis and treatment are mainly in the research stage, which still have some distance from clinical application. In this period of rapid development in the materials sector, relevant research on Pdots needs to constantly combine new techniques and explore more applications. In our opinion, future work in this field mainly includes the following aspects: (1) To develop a large-scale, eco-friendly and low-cost method for the preparation of Pdots. (2) Optimizing biofunctionalization strategies to obtain smart Pdots-based probes with stimuli-responsive targeting. (3) Pdots with ultra-small particles (<10 nm) and uniform particle size that are particularly suitable for the development of optical transducers. Furthermore, for in vivo applications, the ultra-small-size Pdots can further enhance biodistribution and rapid metabolism. The biological metabolism of nanoparticles is currently the biggest problem limiting their clinical application. (4) The exploration of NIR-II Pdots can improve the penetration depth of light in biological tissues. Although NIR-II imaging and therapy have received considerable attention, biosensing applications of the NIR-II window are largely unexplored. (5) Emerging hybrid nanocomposites composed of organic and inorganic nanomaterials are expected to endow the nanosystems of Pdots with complementary multimodal imaging modalities and synergistic therapeutics. (6) Computational simulations can also help us design functionalized Pdots for specific biomedical applications. With the rapid development of artificial intelligence, it will be more effective to design rational Pdots for clinical translation.

## Figures and Tables

**Table 1:** Pdots for biosensing and in vivo imaging.

Pdots	*λ*_max_^abs^ (nm)	*λ*_max_^em^ (nm)	*Φ* (%)	Application	Ref.
DPA-CNPPV	294	627	10.8	NADH sensing	[50]
PD4Gx	380	425, 672	11.5	Glucose monitoring	[51]
PF-TC6FQ	~360	~670	N.A.	PSA detection	[52]
PFCN	~390	~450	N.A.	AFP detection	[52]
PFO	~350	~490	N.A.	CEA detection	[52]
APNs	700	720	N.A.	Cancer and allograft	[53]
ASPNC	~440	680	N.A.	Exosomes sensing	[54]
NIR MEH-PPV	504	776	N.A.	Fluorescent imaging	[55]
m-PBTQ4F	946	1123	3.2	Fluorescent imaging	[56]
RET_2_IR NPs	503	778	0.18	Fluorescent imaging	[57]
Pdots-C6	745	1055	N.A.	Fluorescent imaging	[58]
SPN-PT	1064	N.A.	N.A.	Photoacoustic imaging	[59]
DPP-BTzTD	~1000	N.A.	N.A.	Photoacoustic imaging	[60]
SPNs	~490	780	N.A.	Afterglow imaging	[61]
SPPVN	500, 775	775	51.0	Afterglow imaging	[62]
SPN-NIR	452, 773	507, 775	2.12	Chemiluminescent imaging	[63]
SPNRs	450, 460, 580	520, 540, 700	2.7 ± 0.014	Chemiluminescent imaging	[64]
rSPN2	~680	840	N.A.	Multimodal imaging	[65]
PPE Gd-SPNs	388	440, 470	22.0	Multimodal imaging	[66]
Au-NP-Pdots	525	~440, 460	18.0	Multimodal imaging	[67]

N.A.: Not applicable.

## Data Availability

Not applicable.
